# A Dance to the Music of Time: Aesthetically-Relevant Changes in Body Posture in Performing Art

**DOI:** 10.1371/journal.pone.0005023

**Published:** 2009-03-26

**Authors:** Elena Daprati, Marco Iosa, Patrick Haggard

**Affiliations:** 1 Dipartimento di Neuroscienze and Centro di Biomedicina Spaziale, Università degli Studi di Roma Tor Vergata, Roma, Italy; 2 Dipartimento di Fisiologia Neuromotoria, IRCCS Fondazione Santa Lucia, Roma, Italy; 3 Dipartimento di Scienze del Movimento Umano e dello Sport, Istituto Universitario di Scienze Motorie, Rome, Italy; 4 Institute of Cognitive Neuroscience, University College London, London, United Kingdom; University of Cambridge, United Kingdom

## Abstract

In performing arts, body postures are both means for expressing an artist's intentions, and also artistic objects, appealing to the audience. The postures of classical ballet obey the body's biomechanical limits, but also follow strict rules established by tradition. This combination offers a perfect milieu for assessing scientifically how the execution of this particular artistic activity has changed over time, and evaluating what factors may induce such changes. We quantified angles between body segments in archive material showing dancers from a leading company over a 60-year period. The data showed that body positions supposedly fixed by codified choreography were in fact implemented by very different elevation angles, according to the year of ballet production. Progressive changes lead to increasingly vertical positions of the dancer's body over the period studied. Experimental data showed that these change reflected aesthetic choices of naïve modern observers. Even when reduced to stick figures and unrecognisable shapes, the more vertical postures drawn from later productions were systematically preferred to less vertical postures from earlier productions. This gradual change within a conservative art form provides scientific evidence that aesthetic change may arise from continuous interaction between artistic tradition, individual artists' creativity, and a wider environmental context. This context may include social aesthetic pressure from audiences.

## Introduction

Artistic culture appears to be a uniquely human attribute. Although art exists in many forms, three characteristic features are commonly present. These include a creative artist, an “art object”, and an observer or audience. The combination of the first two features may result in a particular class of psychological experience in the observer, which has been called ‘aesthetic experience’ [1].

Here we explore the relation between individual aesthetic experience and changes in art objects over extended period of time. The nature of aesthetic experience, and its neural basis, have both been hotly debated. One tradition has sought more or less universal principles of aesthetic experience. For example, simple geometrical relations were proposed to explain preferences for some abstract shapes above others [2]–[4]. However, these effects are often small and far from universal. Even the best known, such as the ‘golden section’ [5]–[6] have proved difficult to replicate [7]. An alternative viewpoint questions the validity of aesthetic universals, and instead points to the clear and important role of culture in aesthetic evaluation. For example, aesthetic preferences might result from an extension of the laboratory effect of mere exposure [8], whereby we come to like what is familiar [9]. However, since this view reduces aesthetic experience to repetition, it predicts that artistic culture should be rather static. It cannot account for changes in artistic production, nor for the role of individuals' aesthetic experience in such change. In fact, artistic culture often involves a balance between the traditions (“schools”) of a particular art form, and innovation or change. The role of artistic tradition may explain why some art forms are gradually perfected over time, achieving potent and lasting effects on individuals and even whole societies. The role of innovation may explain why mimicry alone is not generally considered artistic.

Changes in artistic expression arise from the creative processes of the individual artist, but also reflect a wider environmental context, such as enabling technologies, audience feedback, and market forces. In performing arts, factors related to the artists' fitness, motor capabilities and training would also be an issue. The dynamics of this balance between tradition and innovation have often been described by art historians [10]. Interestingly, however, they have received much less attention from scientists, despite a clear similarity to general principles of exploitation vs. exploration that underlie evolution of individual and group behaviors [11]. One reason for the lack of scientific attention may be that artistic change is often unconstrained, and difficult to quantify, reflecting the fact that human creativity appears infinite [12]. However, in some cases, changes are more subtle and can be objectively quantified, for example because change occurs within a strong artistic tradition, or because the raw material is intrinsically limited. Such cases provide a benchmark for a scientific understanding of how and why artistic cultures may change and develop.

Classical ballet offers one such case, as it involves quantifiable raw material in the form of human body postures, a strong and clearly defined artistic tradition, and rich objective documentation in the form of archives. These conditions allowed us to focus on changes in small number of quantifiable variables, repeated across different individual artists, and spanning a suitable period of time. Ballet is a dance form, which uses a single, codified, set of positions of the human body to express emotions and other mental states. Because the positions of the human body are biomechanically constrained, the artistic raw material is limited. Classical ballet is traditional and conservative: dancers today use the same positions that were codified in 1760 [13], and often follow choreographies established in the 19th century. The body positions appropriate at each moment in the ballet are transmitted between generations within ballet companies by observational learning and instruction [14], by books of notation [15] and more recently by video records. More importantly, body positions can be easily measured from the archive material, providing a suitable database for scientific investigation.

This combination allowed us to compare successive artistic interpretations of a fixed piece of choreography. Specifically, we tested the hypothesis that performing arts show a slow, progressive change that parallels the changing aesthetic preferences of audiences. For this purpose, we explored whether successive dancers have modified the expression of standard body postures, despite the codified rules of classical dance, and the constraints imposed by the laws of biomechanics. Because dance aims to produce aesthetically relevant visual patterns using the dancer's body as raw material, such variation could either reflect changes in dancers' flexibility and muscular strength, or could reflect changing aesthetic references. We tested this second possibility in an additional study by investigating the aesthetic impact of the visual patterns produced by the dancers' bodies in our dataset.

## Results

### Study 1: Tracing progressive changes in body postures

**Figure 1:**
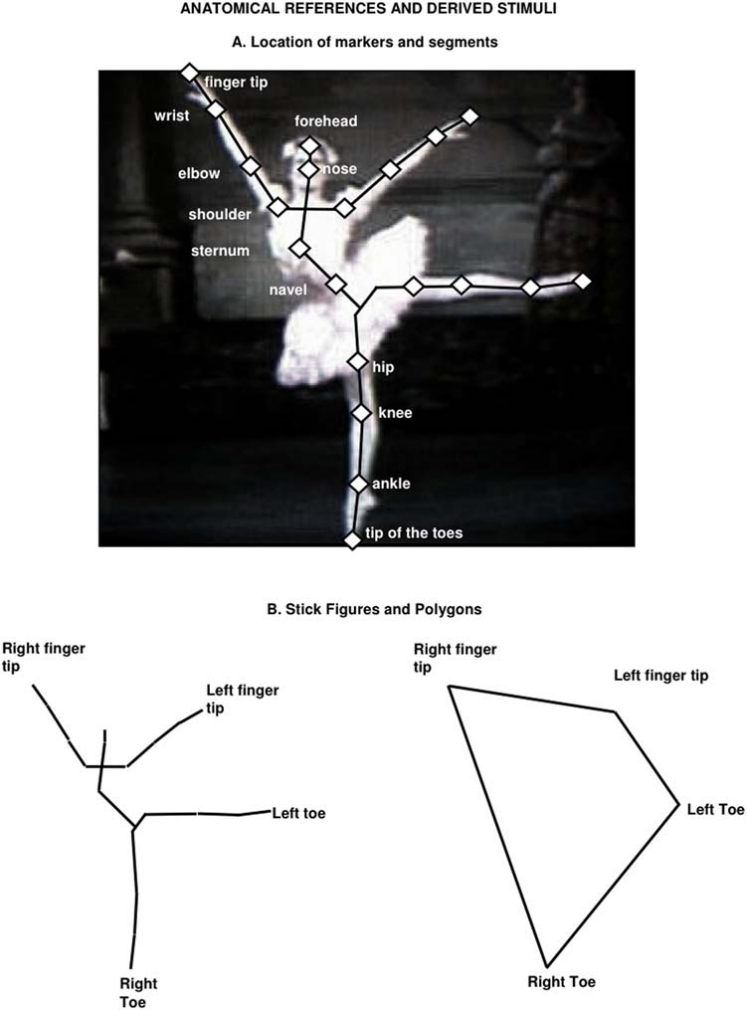
Anatomical references for angle analysis and stimuli for the aesthetic preference experiment. A. Body-part labelling and joint angle calculation. Three independent judges positioned a digital marker on the major joints and body-parts. Marker locations were used to compute limb and axial segments and to define angles. They were also employed in producing stick figures and polygons. B. Stick figures and geometric shapes used in aesthetic preference task (see text for details). Stick figures were obtained by joining the markers shown in A; polygons were defined by the limb endpoints of the stick figure. The labels on the stick figure and polygon were not present during testing.

Ballet positions are codified, and limited by biomechanics' rules. Theoretically, they should present in identical form across time, except for slight random variations due to variability between individual dancers. Study 1 aimed to investigate whether variation across time in specific ballet positions is indeed random, or whether there is a systematic trend across time. For this purpose, we selected a single piece of choreography from The Sleeping Beauty. This work has been performed in essentially identical form across decades [16]. Within this single piece, we chose canonical, codified body positions that occurred with several consistent repetitions, so that we could average repeated measurements (see Figure 1 and Methods).

We collected photographs and video material from the archive of the Royal Opera House, Covent Garden, covering Royal Ballet productions in the period 1946–2004 (see Methods). We measured joint angles from the archive material, concentrating on the angle of elevation of the leg in six standard body positions (see Methods). Joint angle measures were chosen because they are independent of the dimensions of the image, of the camera-dancer distance, and of the body size and body proportions of the individual dancer. We related the angle of elevation to the year of each production of The Sleeping Beauty and performed separate regression analyses for each selected body position.

**Figure 2:**
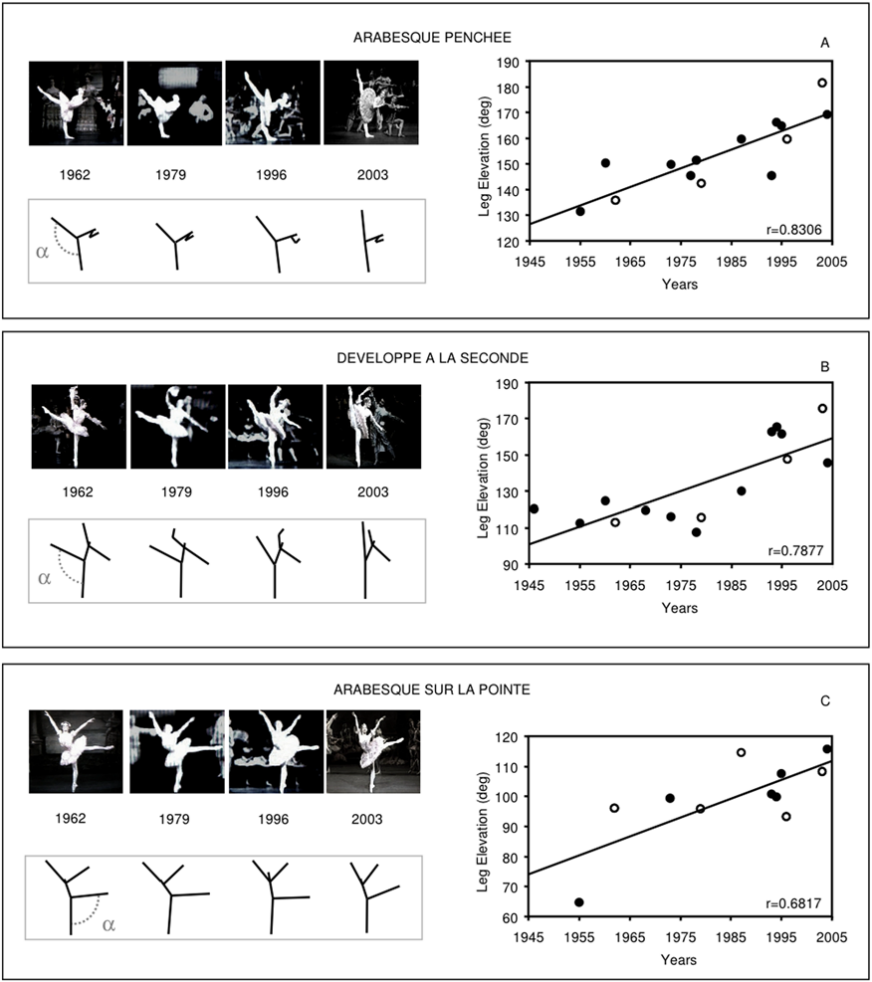
Three representative examples of the body positions analysed. Panel A: A highly skilled, unsupported position. The leg elevation that the dancer can achieve depends on her flexibility and her ability to simultaneously maintain balance; Panel B: A highly skilled, supported position. Balance is made easier by the male dancer's support, and leg elevation depends largely on flexibility; Panel C: A less skilled, unsupported position. The leg elevation required by the choreography is less demanding compared to the posture in panel A and balance is less critical, as the position should be maintained only for a brief time. In each panel: right side - correlation between year (x-axis) and degree of leg elevation (y-axis), r = Pearson correlation coefficient; left side - upper row, archive material showing the positions where the angle was recorded; lower row: stick figures showing the mean angle for the corresponding year. The angles measured from these illustrative images appear as red dots along the corresponding regression line.

Figure 2 shows representative examples of the correlation analyses relating the angle of leg elevation to the year of each production of The Sleeping Beauty for three of the selected body positions (see Table 1 for statistics). Clearly, despite the codified positions established by the choreography, there are systematic differences in elevation angles according to the year of ballet production.

**Table 1:** Correlation analyses for leg elevation and trunk inclination angles vs. year.

I. ANGLE^1^ OF LEG ELEVATION		r	p	N
**Adagio**	1 - Arabesque penchée (HS)^2^^,^ 3	0.831	<0.0002	14
	2 - Développé à la seconde (HS)^2^^,^ 4	0.788	<0.0005	15
	3 - Arabesque roses (HS)^2^	0.871	<0.01	7
	4 - Arabesque sur la pointe (LS)^5^^,^ 6	0.682	<0.01	12
	5 - Piqué arabesque sur la pointe (LS)^5^	0.649	<0.05	9
**Fairy of purity**	6a - Développé à la seconde - right leg (HS)^2^	0.795	<0.02	8
	6b - Développé à la seconde - left leg (HS)^2^	0.797	<0.02	8

II. ANGLE^1^ OF TRUNK INCLINATION		r	p	N
**Adagio**	1 - Arabesque penchée (HS)^2^^,^ 3	−0.137	NS	14
	2 - Développé à la seconde (HS)^2^^,^ 4	−0.420	NS	15
	3 - Arabesque roses (HS)^2^	0.489	NS	7
	4 - Arabesque sur la pointe (LS)^5^^,^ 6	0.155	NS	12
	5 - Piqué arabesque sur la pointe (LS)^5^	−0.751	<0.02	9
**Fairy of purity**	6a - Développé à la seconde - Right Leg (HS)^2^	0.616	NS	8
	6b - Développé à la seconde - Left Leg (HS)^2^	−0.605	NS	8

1 Angular values for the analysed positions were averaged across judges prior to analyses; r = Pearson correlation coefficient; N = number of images available.

2 HS = Highly Skilled postures: postures highly demanding in terms of balance (i.e. the ballerina is not supported by the male dancer) and/or motor constraints (i.e. leg elevation above 90–120 deg).

3 see an example in Figure 1 A.

4 see an example in Figure 1B.

5 LS = Less-skilled postures: postures less demanding in terms of balance (i.e. the ballerina is supported by the male dancer) and/or motor constraints (i.e. leg elevation below 90–120 deg).

6 see an example in Figure 1C.

In the first example (panel A), the angle of the working leg increases progressively with time (see Table 2 for mean values). Since the ballerina is unsupported, this historical variation corresponds to a considerable increase in the skill required to maintain balance, while still producing a defined and expressive geometry of the body. Interestingly, the variability between successive reproductions of a given body position by the same dancer in one production is low, reflecting their high levels of motor skill. In contrast, the variability in average leg elevation across years is relatively high. This suggests that the historical trend cannot be due merely to a sampling bias in the particular images chosen for measuring leg elevation. Table 3 shows an example comparing variability across dancers and variability within a single dancer, for the case of the body position shown in Figure 2A).

**Table 2:** Amplitude of leg elevation angles for the positions included in the study.

Développé à la seconde			Arabesque penchée			Arabesque sur la pointe		
*Year*	*Mean* 1	*SD* 2	*Year*	*Mean* 1	*SD* 2	*Year*	*Mean* 1	*SD* 2
*1946*	120.4	6.7	-	-	-	-	-	-
*1955*	112.6	6.0	*1955*	131.6	4.6	*1955*	64.7	6.1
-	-	-	*1959*	148.7	7.8	-	-	-
-	-	-	*1959*	152.0	6.0	-	-	-
*1960*	124.7	5.5	-	-	-	-	-	-
*1962*	112.9	2.8	*1962*	136.9	13.6	*1962*	96.1	8.3
*1968*	119.6	1.3	-	-	-	-	-	-
-	-	-	-	-	-	*1969*	102.0	9.6
*1973*	115.0	10.6	*1973*	151.4	14.3	*1973*	99.4	7.0
-	-	-	*1977*	133.4	6.2	-	-	-
-	-	-	*1977*	157.2	10.0	-	-	-
*1978*	107.5	5.7	*1978*	160.9	12.0	-	-	-
-	-	-	*1978*	141.7	13.5	-	-	-
*1979*	109.9	2.1	*1979*	142.4	11.1	*1979*	95.8	9.5
*1979*	139.4	9.3	-	-	-	-	-	-
*1987*	128.1	10.2	*1987*	158.0	6.7	*1987*	114.6	14.7
*1993*	162.8	5.1	*1993*	146.2	7.2	*1993*	100.8	21.0
*1994*	165.4	3.0	*1994*	167.6	15.8	*1994*	96.8	7.7
-	-	-	-	-	-	*1994*	107.0	3.8
-	-	-	-	-	-	*1994*	100.6	11.5
*1995*	161.5	1.5	*1995*	164.9	9.0	*1995*	107.6	11.0
*1996*	147.5	3.2	*1996*	161.0	15.5	*1996*	93.2	2.9
*2003*	175.6	2.1	*2003*	181.6	7.5	*2003*	108.4	5.4
*2004*	145.5	4.8	*2004*	169.3	13.7	*2004*	115.7	6.1

Piqué arabesque sur la pointe (right leg)			Piqué arabesque sur la pointe (left leg)			Arabesque roses		
*Year*	*Mean* 1	*SD* 2	*Year*	*Mean* 1	*SD* 2	*Year*	*Mean* 1	*SD* 2
-	-	-	-	-	-	*1946*	131.1	4.8
-	-	-	-	-	-	*1957*	151.6	3.5
*1962*	92.8	8.6	*1962*	99.9	8.6	-	-	-
-	-	-	-	-	-	*1969*	164.7	3.1
*1973*	123.6	7.0	*1973*	93.4	9.2	*1973*	179.1	1.9
*1979*	97.7	11.2	*1979*	97.7	8.5	-	-	-
-	-	-	-	-	-	*1987*	179.2	3.5
*1993*	115.8	4.1	*1993*	94.9	5.9	*1993*	171.9	3.5
*1994*	129.2	2.9	*1994*	105.5	7.1	*1994*	187.9	3.6
-	-	-	-	-	-	*1994*	166.0	6.3
*1995*	129.9	8.3	*1995*	107.7	9.3	-	-	-
*1996*	104.6	4.7	*1996*	98.8	3.7	-	-	-
*2003*	114.0	-	*2003*	117.4	-	-	-	-
*2004*	126.9	8.4	*2004*	111.6	8.8	-	-	-

1 Mean values (averaged across the three judges).

2 standard deviations are reported for the material analysed for each year of the ballet production. Please refer to Table 1 for the number of entries computed for each position.

**Table 3:** Leg elevation data for Arabesque Penchée: angular measurements of leg elevation by three independent judges (J1, J2, J3) for one representative position (shown in Figure 1A).

J1							J2						J3					
*Year*	*1^st^* *	*2nd*	*3rd*	*4th*	*Mean*	*SD*	*1^st^* *	*2nd*	*3rd*	*4th*	*Mean*	*SD*	*1^st^* *	*2nd*	*3rd*	*4th*	*Mean*	*SD*
*1955*	130	133	131	136	132	3	126	120	126	134	127	6	136	136	134	136	136	1
*1962*	129	144	138	143	139	7	120	123	123	123	122	1	142	152	154	-	149	6
*1973*	122	152	140	150	141	14	144	154	148	134	145	8	163	171	169	-	168	5
*1979*	138	140	146	144	142	4	128	129	130	138	131	4	170	154	129	161	153	17
*1987*	159	147	-	-	153	8	155	169	168	159	163	7	-	-	-	-	-	-
*1993*	152	142	143	140	144	5	153	132	142	134	140	9	167	147	148	-	154	11
*1994*	173	171	167	163	169	4	156	155	153	141	151	7	184	181	184	-	183	2
*1995*	170	170	167	165	168	2	158	157	152	151	155	4	176	173	171	-	172	4
*1996*	169	167	156	164	164	6	151	146	139	140	144	6	182	176	166	-	175	8
*2003*	181	172	188	-	180	8	178	166	180	-	175	8	186	184	198	-	190	8
*2004*	160	173	174	-	169	8	155	154	160	-	156	3	184	178	189	-	183	5

* *The position is repeated 4 times within the choreographic excerpt studied, allowing an estimate of intra-individual variability.*

In addition, our analyses show that the increasing leg elevation is a specific change in the movement of the working leg. A biomechanical analysis of this change suggests that the leg is lifted in order to achieve a desired vertical line of the whole body. If the aim were merely to lift the leg as high as possible, greater leg elevation can always be achieved by inclining the trunk towards the horizontal. However, we found that the trunk angle was maintained despite increased leg elevation (see Table 1). Maximal abduction of the working leg is likely to involve greater external rotation of the hip (“turnout”). Several studies emphasize the correlation between leg abduction and hip external rotation [17]–[19]. Moreover, turnout is a basic, yet critical aspect of ballet training: elite dancers tend to show greater hip external rotation compared to controls. We therefore speculate that the effect of increasing leg elevation arose from perfecting this basic building block of ballet body position. By progressively increasing the functional range of movement of the working leg, the dancer is offered the opportunity to produce the pose that best matches the current aesthetic choice: here, making the overall bodyline more vertical (at least across this historical period).

The hypothesis that the progressive change towards more vertical positions is not mere virtuosity or display a dancer's maximal performance ability but rather reflects changing aesthetic choices is supported by the finding that this trend is quite general. It emerges in postures where leg elevation does not approach the maximum values and in dancers of different experience. Thus, figure 2B shows a trend towards increasing elevation in a position where the ballerina is supported by the male dancer. The presence of additional support makes this posture less biomechanically demanding than the posture of Figure 2A. The leg could therefore be lifted higher than it actually is even in older productions. However, the dancer's body tends towards a more vertical line only in the later productions within our time period, even for these positions in which a vertical line was always possible. Similarly, the trend towards verticality is shared by the dancers interpreting the “Fairy of purity”, a short variation traditionally executed by a younger or less experienced dancer (see Table 1).

Taken together, these findings suggest that variations across time in the elevation of the working leg have occurred for iconic moments in classical ballet. These variations may certainly reflect progressive variations in skill level and dancers' physical fitness. However, as the artists differentially make use of this advantage, we suggest these changes may also reflect aesthetic choices. This possibility was directly addressed in Study 2.

### Study 2: Aesthetic relevance of the historical change in body postures

In Study 2 we directly assessed the hypothesis that the reported progressive variation in body positions reflects a change in aesthetic evaluation. We used the established method of aesthetic preference judgment [20]. We transformed our images into a standardized form, by making separate sets of stick figures based on the principal body segments, and quadrilateral shapes, by connecting the endpoints of each limb in each image. The latter were not obviously recognizable as human bodies. Twelve naïve volunteers participated on the basis of written informed consent. Each participant viewed all possible pairings of stick figures, and all possible pairings of quadrilateral shapes for each body position. They judged which image of each pair they preferred. We averaged preferences across participants, to calculate a preference coefficient for each stick figure or quadrilateral shape, and used linear regression to relate our participants' preference of each figure or shape to the year of the production from which each original image was selected. The experiment did not seek to identify any universal aesthetic rules, but only to investigate whether historical changes in artistic production were relevant to the aesthetic experience of our participants. The study procedures were approved by UCL's Department of Psychology ethics committee.

**Figure 3:**
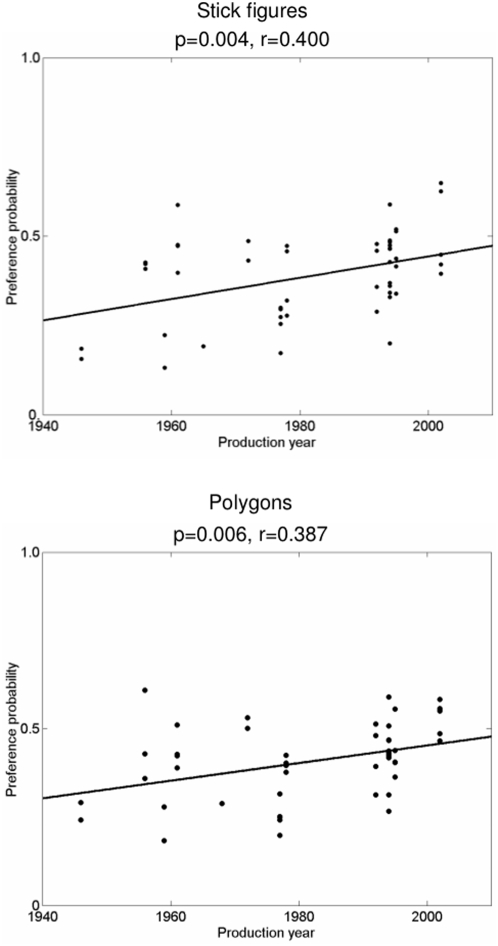
Results for the aesthetic preference experiment. Twelve naïve volunteers (without significant experience of performing or attending dance) viewed all possible pairings of stick figures/ polygons for each body position, and judged which image they preferred. A preference coefficient for each stick figure/ shape was then computed by averaging preferences across participants. This was defined as the probability of each figure or shape being preferred to all the other figures or shapes representing the same body position. Linear regression was applied to relate preference of each figure or shape to the year of the production from which each original image was selected. r and p values for the two analyses are given.

Both the regression analysis for stick figures and shapes were significant (Figure 3). Namely, there was a significant tendency for figures and shapes drawn from later productions to be preferred to those from earlier productions. Besides, the mean of regression slopes for each individual combination of image type (stick figure/shape) and body position had an average significantly greater than zero (p = 0.029), suggesting a general relation between production year and aesthetic evaluation.

Based on these findings, we suggest that the historical variation of body positions shown in Study 1 may reflect changing aesthetic evaluations of body posture. The consensus of our participants showed that greater elevation angles of recent Sleeping Beauty productions are, in general, preferred to lower elevation angles of earlier productions. We found similar preferences even when bodies were transformed into abstract geometric shapes. Preferences for verticality in geometric shapes have been reported previously [4]. Here, the dancers' postures were adjusted to generate a progressively more vertical shape over time. The modifications of body posture in classical dance had implications on the average aesthetic response of our contemporary non-expert participants.

Our statistical design treated participants as a fixed effect, and body postures as a random effect: it therefore generalizes across ballet postures but does not make any attempt to generalize across people. Indeed, psychological studies of aesthetics, which aim at generality across individuals, typically show much smaller effects despite much larger subject numbers [3]. Therefore, we can only speculate on whether our results would generalize to other groups of subjects, or to other historical periods. For example, our result suggests that if we had tested people at the start of our time-period, in 1946, they should have preferred less vertical postures. But this hypothesis seems difficult to test: aesthetic preferences vary within individuals across time as a function of their experiences [8]. Therefore, individuals who preferred less vertical postures in 1946 might nevertheless prefer more vertical ones if tested now, due to the intervening exposure to the increasing verticality we have described. A more fruitful focus for future research might involve comparing aesthetic preference judgments from different cultures, and from individuals exposed to different dance forms [21], since aesthetic preferences vary both across cultural groups, and across individuals within groups [22].

## Discussion

We have found a progressive and generalized change in the body positions used to realize selected choreographic moments in successive productions of a classical dance work. Specifically, across several positions, several individual dancers, and several decades, the working leg is lifted progressively higher. Since classical ballet uses codified positions, and since the productions studied here all used a fixed choreography, elaborated and transmitted by the practice of a permanent ballet Company, this historical variation within a single body position category may seem surprising. The linear trend shows that variations in body position are systematic, not random. Interestingly, although we specifically monitored leg elevation angles, the trend clearly involves the global visual shape produced by the posture. Indeed, dancers have not simply aimed at progressively increased leg elevation per se. That could be achieved by a corresponding change in trunk inclination, but we found no evidence for changes in trunk position. Rather, the dancers appeared to perfect the biomechanics of the movement, perhaps by increasing hip turnout, and consequently the range of movement of the working leg. This achieved the characteristic vertical bodyline of recent productions, without modifying trunk position. From this pattern of evidence, we conclude that the variation in leg elevation is an example of progressive, systematic change within an artistic tradition.

### Fitness and/or aesthetics as causes of systematic change

Many factors influence artistic production. We here consider two possible explanations for the historical changes in our dataset: changing dancer fitness, and changing aesthetic preferences. On the first view, change in body position is driven by increases in dancers' fitness and skill. Improvements in dancer selection, training and physical condition have allowed successive generations of dancers to achieve and even exceed the codified body positions laid down early in ballet tradition [13]. Perhaps the dancers earlier in our period would have produced leg elevations equal to those seen more recently, had they been physically able to do so. That view suggests that dancers have throughout strived for a “perfect” body position corresponding to a universal aesthetic goal. Recent dancers have come closer to achieving this goal due to their greater fitness and skill. This view has difficulty explaining why dance forms differ widely across cultures. An alternative view suggests that aesthetic goals are not fixed, but vary, despite the stabilizing effects of codification and tradition. On this view, historical changes in body position at fixed moments of choreography would reflect secular changes in what body positions are given high aesthetic value by prevailing artistic culture. This evaluation presumably reflects the opinions of directors, audiences and dancers themselves.

Based on the experimental data, four reasons suggest that aesthetic considerations play an important role in this process of change. First, our results show that naïve subjects on average prefer the greater elevations of recent years to lower elevations, even when the surface form is reduced to minimal geometric quadrilaterals. That is, the geometric form made by the dancer's body is aesthetically relevant, at least to the subjects we tested. Second, we found the same historical trend in elevation angle in standard, easily achievable postures (Figure 2C) and in more physically demanding postures (Figure 2A). For example, the dancer in Figure 2C produced an elevation of 96 degrees in 1962, while dancers in later productions increased the elevation to 115 degrees, in 2004. But the same dancer, shown in another position in Figure 2A, was physically capable of achieving an elevation of 137 degrees. This suggests that the lower elevation in the first case represents an artistic choice, rather than a physical limitation. Third, we found similar historical changes in body positions whether the dancer is supported by another dancer or not. Unsupported positions (i.e. Figure 2A) introduce greater constraints of balance, and are thus more limited by the dancer motor skill than supported positions (i.e. Figure 2B). We found the same historical trend for greater elevation in supported positions as in unsupported positions (Figure 2 and Table 3). The historical trend in the case of the easier, supported postures is unlikely to be due to changing levels of motor skill, since these positions are not particularly challenging. Finally, the trend appears to be general, and not due to a few unusual individual dancers. For example, we found the same historical trend in a variation (“Fairy of purity”) traditionally danced by a younger or less experienced dancer, as in the positions of the leading étoile (see also Table 3). Hence, we suggest that this trend may partly reflect changing aesthetic goals across our period, in addition to possible increases in motor skill.

Performing arts clearly involve many aesthetically important elements. In the case of classical ballet studied here, these include narrative, music, costume and interaction with other dancers. We focused on one core feature of dance, namely the dancer using a set of defined body positions as a means of communicating with an audience. The historical change we describe indicates that ballet has become more extreme, at least judged from the particular moments and positions analyzed here. This change corresponds to changes in aesthetic value, not merely in performer ability. According to one recent theory in art psychology, representation of extremes is an essential feature of art [23]. On this view, the artist distorts rather than reflects the source material, so that artistic representations exaggerate characteristic physical qualities of the object. This hypothesis may only partially apply to the present case, as performing arts may not involve representations in the same sense as plastic arts. Indeed, aesthetic communication in performing arts may depend more on motor simulation and empathy, rather than representation [6], [24]–[25]. Simulating a more extreme position might evoke a more intense primary sensory experience in the observer than a less extreme position, and thus produce a greater aesthetic effect.

### Social aspects of artistic innovation

Artistic culture, like other aspects of culture, is affected by a complex set of pressures. These pressures include the creative thought of individual artists, the availability of raw materials, wider social context and the ability to induce aesthetic experience in its audience. Changes in artistic culture could either lead or follow a simultaneous modification of observers' aesthetic perception. A common historical view of art emphasizes the innovative or revolutionary creativity of individual minds, often highlighting the more mimetic production of other artists [26]. This model of creativity might produce stochastic change or oscillation, but would not produce the linear progression across time we have observed. Nor can it explain our finding that successive individuals participated in a common long-term artistic trajectory. Our data suggest an additional, secular form of change in artistic activity that would be neither mimetic nor revolutionary, but characterized by sustained, progressive development, and operating at a supra-individual level. For example, it has been suggested that cultural transmission follows similar processes of variation and selection to genetic transmission [27]. In this context, it would be interesting to know whether the preference for more vertical postures seen in our modern subjects would also have been present had the same stimuli been shown to participants at the start of the time period. An account of aesthetic change based on social feedback between artist and audience would predict preference for less vertical postures at that time.

In conclusion, our study used scientific methods to investigate artistic outputs, with the aim of better understanding how and why those outputs may change over time. We scientifically quantified a pattern of variation within a single dance work over a period of decades, and showed that these changes have a trajectory. An experimental study with non-dancers showed that this trajectory is aesthetically relevant, even when the artistic object is reduced to a simple geometric form. This result indicates that cultural changes in art are not necessarily dramatic consequences of individual inspiration, but can arise from a gradual processes operating at supra-individual levels. These may include continuous social feedback between successive generations of artists and their audiences, and may progressively affect even basic aspects of motor control. Finally, at a time of increasing interaction between science and art, our work makes the strong and timely methodological point that artistic culture can be studied scientifically. Artistic culture, like other human behaviors, is dynamic, measurable, and rooted in human sensory and motor experience.

## Materials and Methods

### Study 1

#### Selection from archive material

We chose a single piece of choreography from Marius Petipa's version of The Sleeping Beauty, namely “The Rose Adagio” of Act I that has been performed in essentially identical form across decades (the Sergeyev Collection from Harvard University Library holds the notated ballet, written according to the Stepanov method of dance notation, [8]). The Sleeping Beauty also contains several short pieces danced by solo dancers in secondary roles. We included one such piece in the study, namely Variation n. 1 (“The Fairy of Purity”) from the Prologue of the ballet, to assess whether change across time was general to the performing art as a whole, or merely reflected the peak performance of a few outstanding artists. The data were taken from the archive of the Royal Opera House, Covent Garden, and covered Royal Ballet productions in the period 1946–2004 (see Table 4). The final selected material for the Rose Adagio included 153 images obtained from 11 video-recordings plus 14 photographs (see Table 4), while for the Fairy Variation there were 47 images derived from 8 video recordings plus 2 photographic images. This dataset therefore provides a representative record of the progression of classical dance during this period.

**Table 4:** Images from the ballet were obtained from the archive of the Royal Opera House, Covent Garden, and covered Royal Ballet productions in the period 1946–2004; final material for the Rose Adagio included images obtained from 11 video-recordings plus 14 photographs.

PHOTOGRAPHS			
N^1^	Year^2^	Dancer	Positions^3^
1	1946	Fonteyn	D
1	1960	Beriosova	D
1	1960	Beriosova	AP
1	1960	Beriosova	AP
1	1960	Horrain	A
1	1968	Sibley	D
1	1977	Kirkland	AP
1	1977	Platel	AP
1	1978	Park	AP
1	1978	Park	AP
1	1978	Park	D
1	1978	Park	D
1	1994	Bussell	A
1	1994	Bussell	A
**VHS**			
14	1961–62	Park	4 (AP/D/A/AS/R)
14	1978–79	Park	4 (AP/D/A/AS)
7	1986–87	Kirkland	3 (AP/D/A)
13	1992–93	Bussell	4 (AP/D/A/AS/R)
14	1994	Durante	4 (AP/D/A/AS/R)
14	1995–96	Mason	4 (AP/D/A/AS/R)
**DVD**			
10	1955	Fonteyn	3 (AP/D/A/R)
13	1972–73	Jenner	4 (AP/D/A/AS)
14	1995	Durante	4 (AP/D/A/AS/R)
13	2002–03	Bussell	4 (AP/D/A/AS/R)
13	2003–04	Marquez	4 (AP/D/A/AS/R)

1 *N” indicates the number of available images derived from each source.*

2 *Year or season.*

3 *Positions analysed: D: développé à la seconde; A: arabesque sur la pointe; AS: piqué arabesque sur la pointe; AP: arabesque penchée; R arabesque roses; the numeral indicates the number of images that were examined for each position.*

Within both solo pieces, we selected canonical, codified body positions that occurred with several consistent repetitions, so that we could average repeated measurements. In particular, we chose positions in which the dancer maintains static balance on one leg while producing complex geometry with the other limbs, i.e. a common icon of classical ballet (see Figure 1 and Table 5). In such postures, the angle of elevation of the working leg relative to the standing leg is related to both to the skill of the dancer, and to the audience's perception. For example, variation in leg elevation angle would produce different geometric configurations of the body that might in turn have different aesthetic effects on the audience. Importantly, elevation angles can be compared across individuals with different body shapes, and can be reliably extracted from archival material.

**Table 5:** Description of the positions included in the study: it should be noted that these postures, firstly established by Marius Petipa for The Sleeping Beauty in 1890, are fixed moments in the choreography and have been performed in identical form for over a century.

ACT 1, SCENE II: “THE ROSE ADAGIO”				
Description	Position	Working leg	Entries^1^	Timing^2^
A movement in which the working leg is drawn up to the knee of the supporting leg, then smoothly extended in the air. This movement is repeated up to 4 times, along a diagonal line: in each pose, the hand of one of 4 male dancers supports the ballerina.	*développé à la seconde (D)*	Right	4	30 s
A pose in which the ballerina stands on one leg; the other leg is extended to the back, in the air. The supporting leg is straight and on the tip of the toe. This movement is performed twice and in both cases, it follows a series of 4 positions where the ballerina maintains balance on the tip of one toe. This pose ends each series of such balances.	*arabesque sur la pointe (A)*	Left	2	1 m & 5 m55 s
A step directly on the tip of the toe, where the ballerina stands on one leg, the other leg being extended to the back, in the air. The supporting leg is straight. This movement is repeated up to 4 times, twice along a rightward diagonal and twice along a leftward diagonal: the ballerina is supported by each of 4 male dancers in turn.	*piqué arabesque sur la pointe (AS)*	Right & Left	4	1 m30 s
A pose in which the ballerina stands on one leg; the other leg is extended to the back, in the air. The supporting leg is straight, the foot is resting flat on the floor and the torso is tilted forward. This movement is performed up to 4 times along a diagonal line.	*arabesque penchée (AP)*	Right	4	2 m10 s
A pose in which the ballerina stands on the tip of the toe with one leg; the other leg is extended to the back, in the air. The supporting leg is straight and the dancer holds a bouquet of roses.	*arabesque roses (R)*	Right	1	3 m30 s

PROLOGUE, VAR. N.1 “THE FAIRY OF PURITY”				
Description	Position	Working leg	Entries^1^	Timing^3^
A movement in which the working leg is drawn up to the knee of the supporting leg, then extended in the air. This movement is repeated up to 6 times, in rapid succession, alternating the right and left leg and/or with the right leg only (depending on the dancer).	*développé à la seconde (D)*	Right & Left	6	50 s

1 *Number of repetitions of the same pose by the dancer in one production.*

2 *The ballet lasts approximately 6 min; timing is given with respect to the beginning of the first movement.*

3 *The ballet lasts approximately 1 min; timing is given with respect to the beginning of the first movement.*

Selection of images was made by a researcher naïve as to the main hypothesis (but with experience as ballet dancer and good knowledge of “The Sleeping Beauty”). To avoid biases in the selection, any archived images corresponding to precise moments in the choreography of the Rose Adagio and Fairy variation were collected (see Table 5 for details). For images obtained from videos, the researcher selected the frame in which the movement (i.e. lifting one leg) reached its maximum before inverting direction. For photos, the researcher included only images where the movement could be clearly identified within the choreographic sequence.

#### Data extraction

All images were converted to black and white pictures and displayed with an identical size (640×480 pixels) on a computer screen. Three independent judges were required to indicate the location of the major joint centers and body-parts on the dancer's body for each image (see Figure 1A), by positioning a digital marker on the picture using a computer mouse. Once one digital marker was placed, dedicated software recorded its 2-D position (x, y) in a reference system, directly derived from the picture. For the upper limb, markers were placed on the fingertips, wrist and elbow joint and on a conventional point on the shoulder (defined as the point on the longitudinal axis of the arm closer to the shoulder). For the lower limb, markers were placed on the tip of the toes, the ankle and knee joints, and a estimated point on the hip (defined as the point on the longitudinal axis of the thigh closer to the hip). On the body axis, markers were placed on the sternum and the navel, using the neckline and waistline of the costume as reference, and on the forehead and nose to map the head location. An example is shown in Figure 1.

Marker locations were used to draw segments for limbs and body axis, which were later employed in producing stick figures and polygons (see Figure 1B, Study 2), and in computing major angles. Three segments were drawn for each limb, by joining the individual markers, as follows: a) upper limb: arm (shoulder-elbow), forearm (wrist-elbow), hand (wrist- fingertip); b) lower limb: thigh (hip-knee), leg (knee-ankle), foot (ankle-toe); c) body axis: head (forehead-nose); upper body (nose-sternum); trunk (sternum-navel).

#### Joint angle computation

We measured joint angles rather than limb positions, because angles do not depend on the dimensions of the image, nor the distance at which picture was taken, or the body size of the dancer. They can therefore be compared across images and across individuals. We computed either the angle defined by two adjacent segments, or the angle defined by the inclination of one segment on the horizontal plane (parallel to the plane of the floor), as appropriate for the body position being studied. Lower limb angles were identified by the slope of the linear fitting on the hip, knee and ankle joint. Angle of leg elevation was defined as the difference between the two lower limb angles. Angle of the trunk was estimated based on inclination of the segment sternum-navel on the horizontal plane.

A preliminary analysis was run on each image to check that the dancer's body was positioned in a suitable plane (i.e. frontal view, or side view), which would allow us to calculate joint angles without confounds introduced by perspective distortion. We first measured the length in the image of corresponding limb segments from the left and right sides of the body. Separate paired t-tests were performed for each picture to compare the lengths of multiple segments between left and right sides: a perspective distortion should lead to a significant difference between sides. Pictures where a significant difference was found were excluded from the analyses (less then 10%).

#### Consistency across judges and reliability of angular measures

Of the three judges, judge 1 and 2 had experience of motion capture and kinematic analysis, while judge 3 was a former dancer. Consistency in angle measurements for all analyzed body positions was assessed by an analysis of covariance. Year was used as independent variable, angle as dependent variable and judge as an extraneous variable (covariate). A significant effect of year (p<0.01) emerged for all positions, but no interactions between year and judge were found (all p>0.05, as described in detail below). In fact, the slopes of linear regression between inclination angle and year of production were very similar for the 3 judges: (0.7, 0.8 and 0.6). Moreover, this close agreement between judges was general across the postures studied. For the développé à la seconde slopes for the judges were 1, 1.1, 0.9 (for the interaction between judge and year: p = 0.9407), for the arabesque sur la pointe 0.58, 0.49, 0.56 (p = 0.9408), and for the piqué arabesque sur la point 0.40, 0.58, 0.46 (p = 0.8164). The absence of a significant interaction effect between year and judge shows that any variation in joint angle over time was not affected by differences in judges' measurements. Given this strong agreement, we averaged data from the three independent judges for subsequent analyses.

#### Statistical analyses

Separate Pearson correlation analyses were used to evaluate whether angles varied linearly across time. When more than one image was available for the same year, these entries were averaged prior to analysis. Regression lines were also computed for angle of trunk inclination.

### Study 2

#### Participants

Twelve volunteer subjects (aged 19–21, 3 females) were recruited for this experiment, on the basis of written informed consent, and in accordance with the local ethical committee guidelines and Declaration of Helsinki. The study was approved by UCL Psychology Department's Ethical Committee. One subject had attended recreational ballet classes for 1 hour/week for some years; the others had negligible previous experience of dance performance or attendance.

#### Stimuli

We created stick figures by connecting individual markers for images in which all the relevant body segments were clearly visible. Fifty images from the original pool responded to this criterion; they showed body positions in Table 5A, and dated from 1946–2002. Material in which movements of the upper arm produced partial blurring of video images was excluded. The original archive images were processed as follows to give a consistent stimulus set. First, stick figures were drawn from each image by connecting the markers described in Study 1 (see Figure 1B, left panel). The stick figures were then standardized to a common height, preserving aspect ratio, and skeletonized to have 1 pixel line width. Each stick figure was further processed using a convex hull technique, to derive a quadrilateral shape by connecting the endpoints of the 4 limbs (see an examples in Figure 1B, right panel).

#### Procedure

The experiment consisted of 10 blocks, one for each combination of 5 body positions measured, and 2 image types (figures, shapes). Blocks were performed in random order. In each block, subjects saw each figure/shape paired with each other figure/shape. Each possible pairing occurred twice, with the order of the members of the pair differing on each occasion. One pairing was viewed on each trial, and the order of pairings was randomized anew for each subject. On each trial, subjects first viewed a central fixation cross for 2000 ms. The first figure/shape of the pair was next presented for 1000 ms, followed by the fixation cross for a further 500 ms, followed by the second figure/shape of the pair for 1000 ms, followed again by the fixation cross. Subjects then made unspeeded responses using the ‘1’ and ‘2’ keys on a keyboard to indicate whether they preferred the first or second figure/shape of the pair.

#### Aesthetic dependent variables for data analysis

We calculated the number of times each stick figure/shape was preferred to the other stick figures/shapes showing the same body position, with which it was paired. This was expressed as a probability, and simply averaged across the 12 participants. The resulting Preference probability expresses a consensus of those participants about each figure/image (“consensus preference rating”). The analysis reflects the preferences of these participants, and is not intended to allow results to be statistically generalized to the population at large. Participants are therefore treated statistically as a fixed effect, rather than a random effect, and the unit of observation is the image, rather than the individual participant [28]. Whether or not these participants' aesthetic preference judgments generalize to the population is outside the scope of this study. The consensus preference ratings for each stick figure/shape were related to the year of ballet production using linear regression. One regression was performed for stick figures, pooling preference probabilities for all 5 body positions, and another for shapes. We also performed 10 individual regressions, for each combination of image type (stick figure/shape) and body position, and tested whether the mean of the 10 regression coefficients differed from 0, using the student t-test.
